# A new compact adenine base editor generated through deletion of HNH and REC2 domain of SpCas9

**DOI:** 10.1186/s12915-023-01644-9

**Published:** 2023-07-11

**Authors:** Yuqiang Qian, Di Wang, Wenchao Niu, Ding Zhao, Jinze Li, Zhiquan Liu, Xun Gao, Yang Han, Liangxue Lai, Zhanjun Li

**Affiliations:** 1 Key Laboratory of Zoonosis Research, Ministry of Education, College of Animal Science, Jilin University, Changchun, 130062 China; 2 CAS Key Laboratory of Regenerative Biology, Guangdong Provincial Key Laboratory of Stem Cell and Regenerative Medicine, South China Institute for Stem Cell Biology and Regenerative Medicine, Guangzhou Institutes of Biomedicine and Health, Chinese Academy of Sciences, Guangzhou, 510530 China

**Keywords:** CRISPR/Cas9, Base editor, ABE8e, Reduce size, AAV

## Abstract

**Background:**

Adenine base editors (ABEs) are promising therapeutic gene editing tools that can efficiently convert targeted A•T to G•C base pairs in the genome. However, the large size of commonly used ABEs based on SpCas9 hinders its delivery in vivo using certain vectors such as adeno-associated virus (AAV) during preclinical applications. Despite a number of approaches having previously been attempted to overcome that challenge, including split Cas9-derived and numerous domain-deleted versions of editors, whether base editor (BE) and prime editor (PE) systems can also allow deletion of those domains remains to be proven. In this study, we present a new small ABE (sABE) with significantly reduced size.

**Results:**

We discovered that ABE8e can tolerate large single deletions in the REC2 (Δ174-296) and HNH (Δ786-855) domains of SpCas9, and these deletions can be stacked together to create a new sABE. The sABE showed higher precision than the original ABE8e, with proximally shifted protospacer adjacent motif (PAM) editing windows (A3- A15), and comparable editing efficiencies to 8e-SaCas9-KKH. The sABE system efficiently generated A-G mutations at disease-relevant loci (T1214C in *GAA* and A494G in *MFN2*) in HEK293T cells and several canonical *Pcsk9* splice sites in N2a cells. Moreover, the sABE enabled in vivo delivery in a single adeno-associated virus (AAV) vector with slight efficiency. Furthermore, we also successfully edited the genome of mouse embryos by microinjecting mRNA and sgRNA of sABE system into zygotes.

**Conclusions:**

We have developed a substantially smaller sABE system that expands the targeting scope and offers higher precision of genome editing. Our findings suggest that the sABE system holds great therapeutic potential in preclinical applications.

**Supplementary Information:**

The online version contains supplementary material available at 10.1186/s12915-023-01644-9.

## Background

The emerging of clustered regularly interspaced short palindromic repeats (CRISPR)-Cas system has revolutionized the field of molecular biology and medicine [1]. Various gene-editing systems have been established based on CRISPR system. Base editors (BEs) can accurately install four types of transition mutations (C-T, G-A, A-G and T-C) on target sequences [2, 3], while prime editors (PEs) can mediate small DNA insertion, deletion, and all 12 base conversions [4]. Recently, CGBE and AYBE have been developed to induce C-G and A-T/C mutations [5, 6]. In contrast to Cas9 nucleases, these gene editors do not generate double-stranded DNA breaks (DSB), do not require a DNA donor template, or do not depend on homologous recombination, making them attractive tools for gene therapy.

Cas9, which is derived from *Streptococcus pyogenes* Cas9 (SpCas9), is the most commonly used nucleases due to its high efficiency and simple NGG PAM requirements. However, the size of SpCas9 (1368 aa) derived gene editor and its sgRNA is too large to be packaged together into a single AAV vector for efficient in vivo delivery. To overcome this bottleneck, Cas9-derived gene editors can be split into two smaller parts through intein-mediated protein trans-splicing [7]. Alternative strategy is to develop more compact Cas9 such as SaCas9 [8], NmeCas9 [9], CjeCas9 [10], St1Cas9 [11], and SpaCas9 [12]. In recent studies, researchers have successfully deleted the RNaseH domain (621 bp) from Moloney murine leukemia virus (M-MLV) reverse transcriptase in the PE system without compromising prime editing activity [13]. To reduce the size of the CBE system, they removed the DNA-binding domain of deaminase PmCDA1 (261 bp) and introduced additional mutations to restore enzyme function [14]. While dCas9 has been shown to tolerate large single deletions of the REC2, REC3, HNH, and RuvCIII domains and still functioning in vitro and in vivo [15], it is still unclear whether BE and PE systems can also tolerate such deletions.

In this study, we found ABE is capable of tolerating substantial deletions of REC2 and HNH domains, whereas CBE and PE systems are unable to tolerate these domain deletions. Furthermore, the sABE achieves base editing both in vitro and in vivo. Compared with ABE8e, sABE enables higher precision, significantly reduced size, and has a PAM-proximally shifted editing window.

## Results

### Single domain deletion of ABE, CBE and PE

**Fig. 1:**
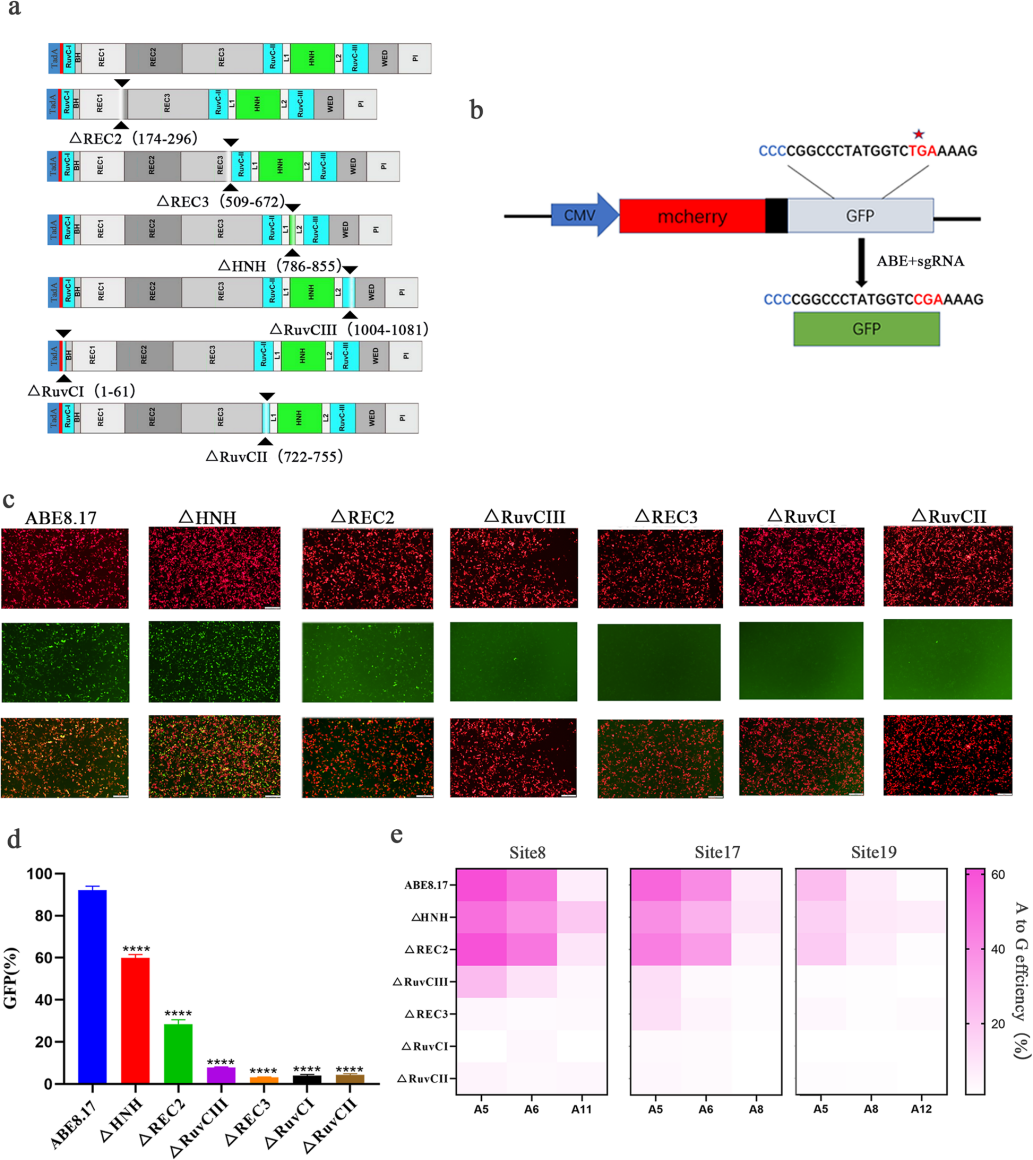
Screening single deletion for ABE. **a** Schematic of domain deletions of ABE8.17. **b** Schematic showing the mCherry/EGFP reporter. **c** Representative fluorescence microscopy images of deletion variants analysis. **d** Comparison of editing efficiency of ABE8.17 deletion variants by flow cytometry analysis (FCA). **e** Comparison of editing efficiency of ABE8.17 deletion variants in HEK293T cell line by plasmid transient transfection (*n* = 3)

To develop reduced size of BE and PE systems, the REC2 (Δ174-296), REC3 (Δ509-672), HNH (Δ786-855), or RuvCIII (Δ1004-1081) domain of AncBE4max [16], ABE8.17 [17], and PE [4] systems were deleted, respectively (Fig. 1a, Additional file 1: Fig. S1a and S2a). Then, the mCherry/EGFP reporters were applied to evaluate the efficiency of these variants [18]. In these reporter systems, ABE and PE with targeted sgRNA or pegRNA were able to convert the stop codon (TGA) to Arg (CGA), and CBE with targeted sgRNA converted Ser (TCA) to Leu (TTA), thus restoring full-length EGFP transcription (Fig. 1b, Additional file 1: Fig. S1b and S2b). The efficiency of gene editing was represented by the fluorescence intensity ratio of EGFP-to-mCherry through flow cytometry (FCM). The mCherry/EGFP assay revealed that AncBE4max could tolerate individual deletion of the four domains with varying efficiencies (Additional file 1: Fig. S1c-d). Moreover, ABE8.17 and PE were able to tolerate deletions of each of the REC2 and HNH domains (Fig. 1c–d and Additional file 1: Fig. S2c-d). In particular, the REC2 deletion variants consistently displayed the highest efficiency among all deletion variants, regardless of the utilization of AncBE4max, ABE8.17 or PE system. However, the mCherry/GFP results could also be influenced by the transfection efficiency and expression efficiency of the editors. Hence, fluorescence report assays are considered to provide only rough assessment of the efficiency of gene editing.

Next, three sites (ABCD, Tp53 and ADAR) were selected to investigate the gene editing capability of AncBE4max variants with domain deletions at endogenous genomic loci. Unfortunately, our results showed that the ΔREC3 and ΔRuvCIII variants failed to edit these three sites, while the ΔHNH and ΔREC2 variants exhibited significantly lower editing efficiency (4 -15%) compared to the original AncBE4max (Additional file 1: Fig. S1e). Recent studies showed ABEs lacking target strand-nicking (nuclease-null dTnpB [19], dCas12f [20] or dCas12j [21]) can efficiently induce endogenous A-to-G conversion, whereas CBEs are unable to induce C-to-T conversion. We thus hypothesized that the lower activity of these AncBE4max variants was due to the lack of target strand nicking of SpCas9 after domain deletion. As expected, we did observe that SpCas9 with REC2 (Δ174-296), REC3 (Δ509-672), HNH (Δ786-855), or RuvCIII (Δ1004-1081) domain deletion abolished DNA cleavage activity (Additional file 1: Fig. S3). Similarly, the PE deletion variants failed to edit endogenous sites of Runx1, Hek2 and Hek3 (Additional file 1: Fig. S2e). Notably, the ΔHNH or ΔREC2 variants of ABE showed comparable efficiency with ABE8.17 at sites 8, 17, and 19 (23.5–60.7% for ABE8.17, 17–50% for ΔHNH and 18.5–60% for ΔREC2) (Fig. 1e). In addition, the ΔRuvCIII of ABE8.17 retained 12–23.5% efficiency for two of the three sites, while ΔREC3 failed to edit any of the three sites (Fig. 1e). To explore further, additional deletion architectures with ΔRuvCI (1–61) and ΔRuvCII (722–755) in ABE8.17 were tested (Fig. 1a), but these variants failed to restore EGFP fluorescence and endogenous editing (Fig. 1d–e). In summary, ABE variants maintained high level of endogenous gene editing, and we thus focus on the domain deletion of ABE for further study.

### Dual-deletion of REC2 and HNH domain forms sABE

**Fig. 2:**
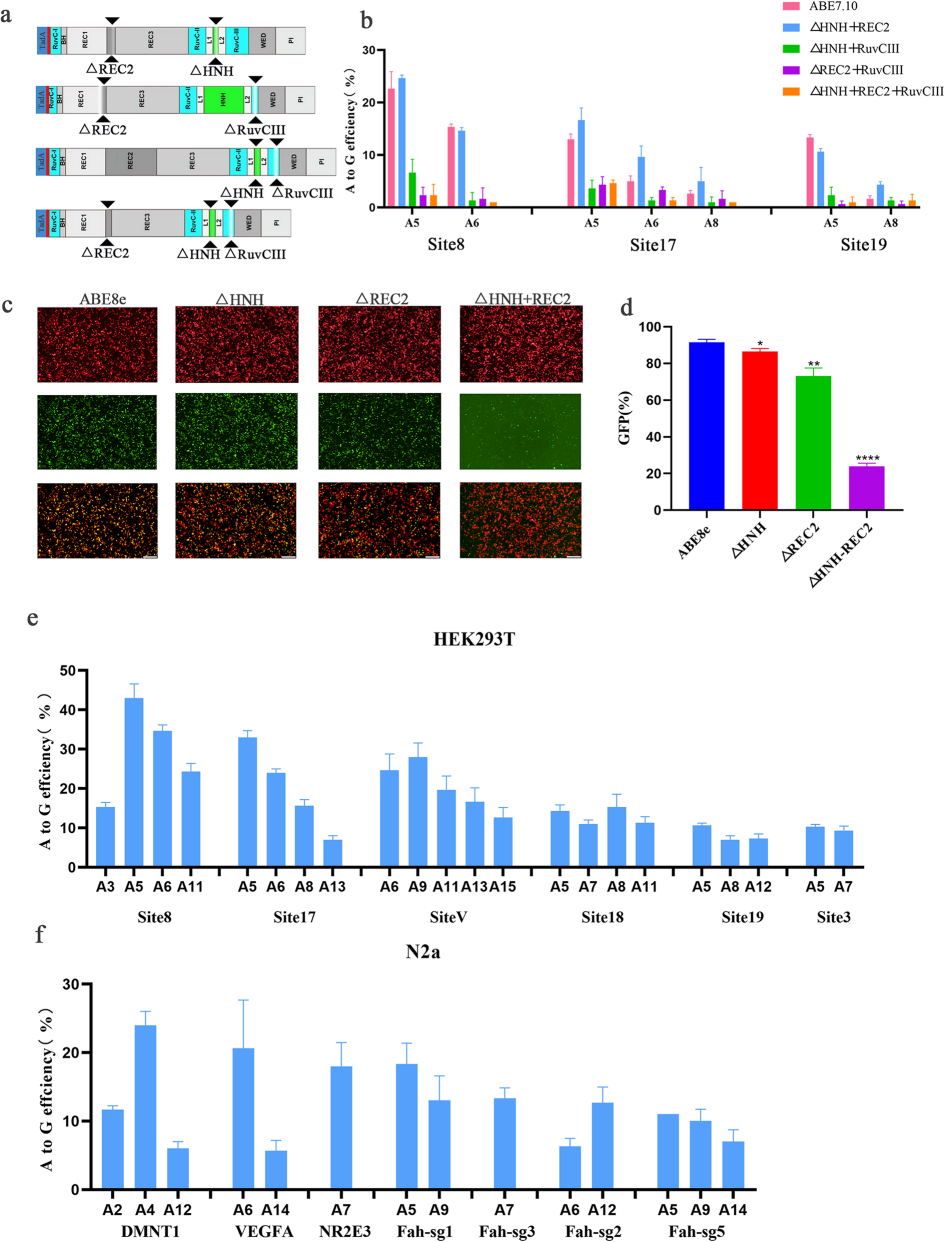
Stacking domain deletions results in sABE. **a** Schematic of multiple domain deletions of ABE8.17. **b** Comparison of editing efficiencies of variants with multiple domain deletions in HEK293T cell line by plasmid transient transfection. **c** Representative fluorescence microscopy images of deletion variants analysis. **d** Comparison of editing efficiency of ABE8e deletion variants by flow cytometry analysis (FCA). **e**, **f** Efficient A-to-G conversions in 6 endogenous loci of sABE in HEK293T cells, and 7 endogenous loci in N2a cells. Error bars indicate SEM (*n* = 3)

ABE8.17 enables the deletion of individual HNH, REC2 or RuvCIII domains while maintaining editing efficiency at endogenous loci. However, it is unknown whether multiple deletions of these domains can result in a further reduction in the size of ABE. Therefore, four possible multiple deletion variants were tested, including three with dual-deletion (HNH + REC2, HNH + RuvCIII, and REC2 + RuvCIII) and one with triple-deletion (HNH + REC2 + RuvCIII) (Fig. 2a). These constructs and corresponding sgRNA were transfected in HEK293T cells. The REC2 + RuvCIII and HNH + REC2 + RuvCIII variants almost completely abolished DNA editing activity, while HNH + RuvCIII exhibited slight editing efficiency of 5–8% at site 8 and site 17 (Fig. 2b). Of note, HNH + REC2 displayed considerable editing efficiency for three sites (8–25%), which was similar to ABE7.10 (Fig. 2b). Therefore, HNH and REC2 domain deletions were selected for subsequent study.

Previous evidence showed that TadA8e outperformed TadA8.17 in rice genome [22]. Therefore, we speculated that TadA8e will enhance the editing performance of ABE variants. As expected, the TadA8.17 replaced by TadA8e in ABE variants resulted in higher efficiency in mCherry/EGFP reporter (ΔHNH, 87 ± 0.9%; ΔREC2, 73 ± 2.53%; respectively). Moreover, the HNH + REC2 variant remained up to 24.9 ± 0.97% of full-length EGFP transcription (Fig. 2c–d). Thus, the optimally small size of ABE8e without REC2 and HNH domain was named sABE.

To thoroughly assess sABE’s efficacy for genome editing, six endogenous loci were targeted in HEK293T cells, sABE exhibiting varied editing efficiencies (10.3–43%) depending on the target site and significantly expanding the editing window (Fig. 2e and Additional file 1: Fig. S4a). Notably, sABE allowed editing up to 12.7% rates at positions 13 and 15 in site 17 and site V (Fig. 2e). We additionally tested sABE at seven targets in N2a cell lines, with editing efficiency ranging from 11 to 24% (Fig. 2f and Additional file 1: Fig. S4b). In summary, we developed an efficient sABE system for introducing gene editing at endogenous genomic loci in both HEK293T and N2a cell lines.

### Characterization of sABE

**Fig. 3:**
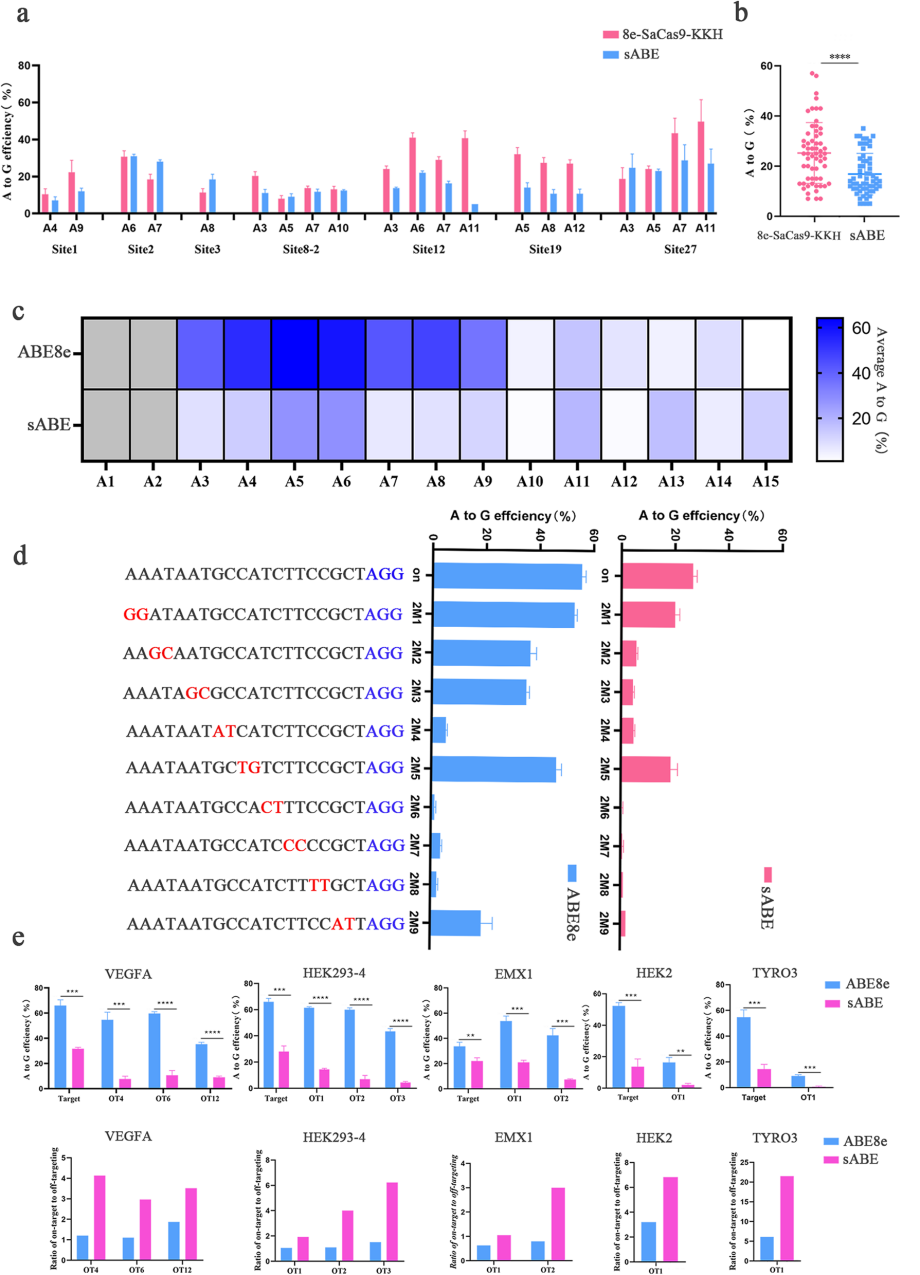
Characterization of sABE. **a** Comparison of editing efficiency in seven target sites using sABE and 8e-SaCas9-KKH (*n* = 3). **b** Summary of the A-to-G editing efficiency induced by sABE and 8e-SaCas9-KKH at the 7 target sites. **c** Summary of editing window for the sABE at endogenous genomic loci. Each data point represents the mean A-to-G editing efficiency at the indicated position of the spacer across 10 target sites, respectively (*n* = 3). **d** Comparison of the tolerance of sABE and ABE8e for mismatched sgRNAs, mismatched sgRNAs that differed from the site 8 by two nucleotides, mismatched nucleotides and the PAM sequence is shown in red and blue, respectively. Error bars indicate the SEM (*n* = 3).** e** DNA off-target analysis comparing sABE and ABE8e plasmid delivery, at VEGFA, HEK293-4, EMX1, HEK2, and TYRO3. Editing efficiencies and on-target: off-target editing ratios are shown

Deletion of the REC2 and HNH domain reduces the size of ABE8e from 4.8 kb to 4.15 kb, which is comparable with the widely used small ABE, 8e-SaCas9-KKH (3.9 kb) [23]. Through side-by-side comparisons in overlapping regions (Additional file 1: Fig. S5), sABE displayed a higher efficiency at sites 2 and 3, comparable efficiency at sites 8–2 and 27, and lower efficiency at sites 1, 12, and 19 (Fig. 3a). Hence, the differences in gene editing efficiency between sABE and 8e-SaCas9-KKH varied depending on the targeted sites, albeit the mean editing efficiency of all selected sites by sABE is lower (Fig. 3b). Similarly, when the deletion of HNH or REC2 domain architecture was adapted to 8e-SaCas9-KKH, we did not obtain substantial A-G editing in the protospacer (Additional file 1: Fig. S6a-b), indicating that SaCas9 can’t tolerant similar domain deletion.

A recent study shows that the removal of HNH nuclease domain expands the editing window because the NHN domain sterically constrains ssDNA accessibility [24]. To define the editing window of sABE compared with ABE8e, we transfected HEK293T cells with plasmids expressing each ABE along with sgRNAs targeting 10 human genomic loci. Although the highest editing rates remained at position A5 and A6, sABE also allowed additional editing at positions 13 and 15 (Fig. 3c). Taken together, these data demonstrate that sABE exhibited a higher editing activity at PAM-proximal adenine.

Anti-CRISPR (Acr) proteins, as a natural brake for CRISPR-Cas technologies, have been widely used to control genome editing in mammalian cells and organisms [25]. To evaluate the inhibition of sABE’s editing activity by Acrs, HEK293T cells were transfected with sABE and sgRNA targeting three sites, along with four known SpCas9 Acr proteins (AcrIIA2, 4, 5, and 16) [26]. The results showed that these Acr proteins also inhibited editing activity of sABE, with AcrIIA5 showing the strongest inhibitory effect (Additional file 1: Fig. S7). This indicates that these Acr proteins may not interact with the HNH and RCE2 domains of SpCas9.

Previous studies have demonstrated that DNA base editing off-target is mainly Cas9-dependent [27, 28], due to Cas9 binding and unwinding at near-cognate sequences. We hypothesized that sABE with truncated HNH and REC2 domain could potentially decrease DNA binding and unwinding. To test this hypothesis, we generated a set of sgRNAs targeting site 8 with dinucleotide mismatches and evaluated their activities through plasmid transfection and sequencing (Fig. 3d). Interestingly, we found that most of the dinucleotide mismatches had a significant impact on the editing efficiency of sABE, whereas ABE8e was able to efficiently edit these mismatches (Fig. 3d). Hence, sABE showed a significantly lower off-target editing efficiency propensity compared to ABE8e. Five previously reported Cas9-dependent off-target sites of VEGFA, HEK293-4, EMX1, HEK2, and TYRO3 were used to analyze off-target activity of sABE in HEK293T cells. We observed a decrease in editing at all on-target and off-target sites when comparing sABE to ABE8e, but the ratio of on-target to off-target editing increased up to four times (Fig. 3e and Additional file 1: Fig. S8). At the HEK2 and TYRO3 sites, we observed up to 14% editing efficiency but failed to generate detectable editing at the off-target sites (Fig. 3e). These data indicate that the sABE system reduced Cas9-dependent off-target editing compared to the original ABE8e.

### Application of sABE

**Fig. 4:**
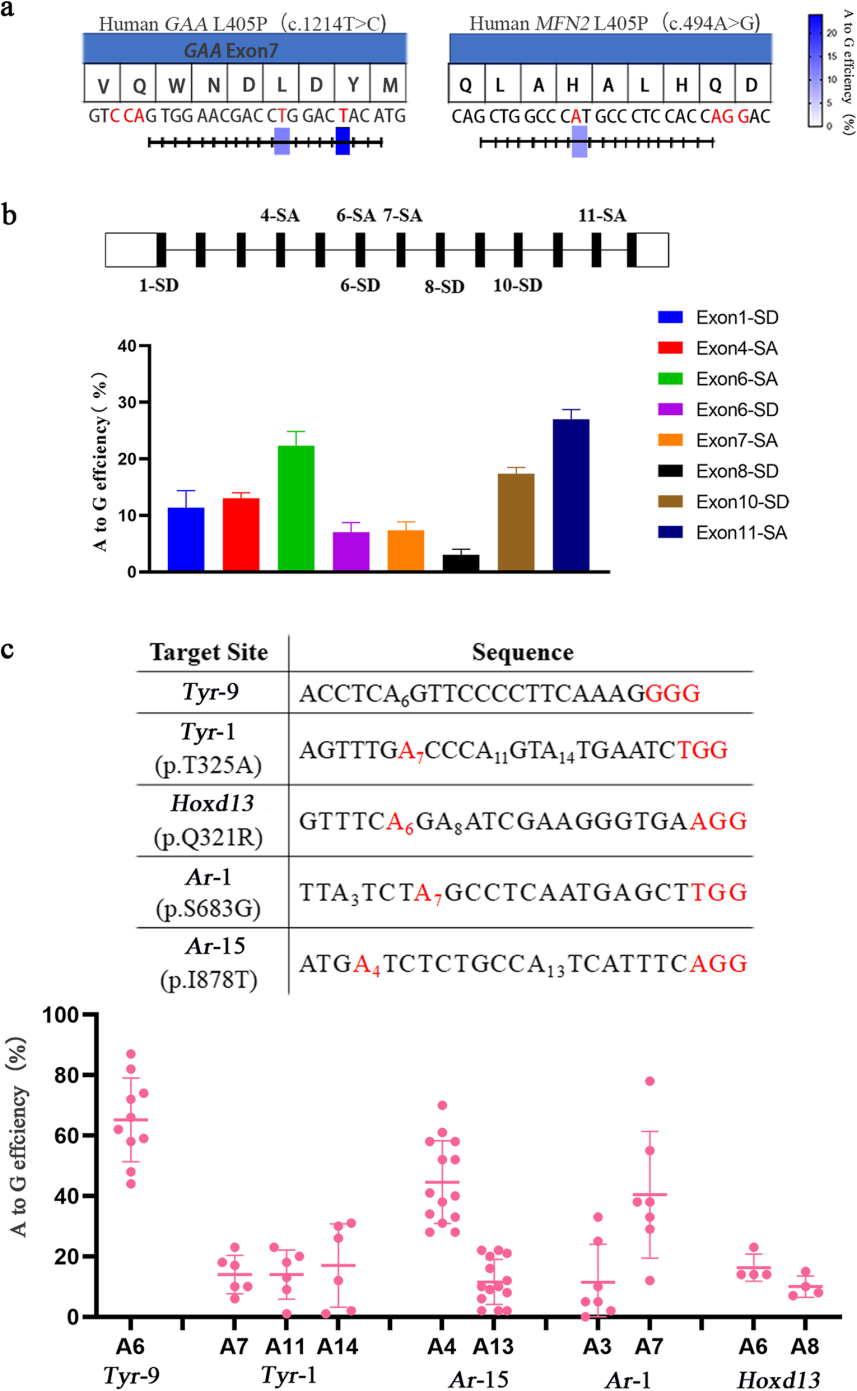
Successful installation of mutations by sABE in cells and mouse embryos. **a** Schematic illustrates the design of sgRNAs for installation. sgRNAs with detectable editing in Sanger chromatogram are displayed. Editing efficiency was detected by Sanger sequencing and shown by Heatmaps. **b** Overview of tested sgRNAs at their respective target locus in murine *Pcsk9* and correlated editing efficiency of splice-donor (SD) sites and splice-acceptor (SA) sites. **c** Target-site sequences within the targeted loci in mouse embryos, and the A-to-G editing frequencies at the target site using sABE. Target sequence (black), PAM region (red) and target sites (red)

Subsequently, we applied sABE to introduce mutations related to glycogen storage disease (T1214C in *GAA*) and Charcot-Marie-Tooth disease (A494G in *MFN2*) in HEK293T cells. We observed successful installation of the two mutations at their respective sites at protospacer position A9 with editing efficiency ranging from 10 to 12% (Fig. 4a). Adenine base editors were utilized to induce targeted A-G editing in DNA at the conserved splice-site motif and inactivate genes [17]. Hence, sABE was used to target several canonical *Pcsk9* splice sites, including splice-donor (SD) sites and splice-acceptor (SA) sites, in N2a cells. The results showed that three of the gRNAs used demonstrated a relatively high level of editing activity (17.3–27%) at the target splice site (Fig. 4b).

Subsequently, sgRNA of Pcsk9 exon 6-SA site was designed for in vivo experiments in mice. We packaged a 180 bp miniCMV promoter, 4.15 kb sABE, 49 bp short translation terminator, and 363 bp U6-driven sgRNA into a single AAV vector, resulting in a 4.83 kb expression cassette (Additional file 1: Fig. S9a). AAV viral particles were injected via tail vein at a dose of 4 × 10^12^ genome copies (GCs) per mouse, with PBS as a negative control. After 1 month, liver tissues were harvested from sacrificed mice. The editing efficiency achieved in the liver of one mouse was only 1.09% (Additional file 1: Fig. S9b). These findings suggest that further optimization is required for AAV-mediated delivery of sABE.

To assess the feasibility and efficiency of sABE in mice, we selected five target sites from three genes (*Tyr*, *Hoxd13*, and *Ar*). After microinjecting of sABE mRNA and gRNAs into mouse zygotes, the zygotes were cultured in vitro and blastocytes were collected individually for genotyping. Our results showed high editing efficiencies of sABE ranging from 23 to 66% at all tested sites (Fig. 4c, Additional file 1: Fig. S10 and Table S1). Thus, the sABE variant is a valuable addition to the ABE toolkit.

## Discussion

In this work, we demonstrate that deleting both the HNH (Δ786-855) and REC2 (Δ174-296) domains of SpCas9 can reduce its size from 4.1 kb to 3.4 kb. The resulting sABE is similar in size to 8e-SaCas9 and can be delivered in vivo using a single AAV vector with minimized promoters. We also found that sABE has an expanded editing window (A3-A15). Moreover, the on-target activity and the off-target propensity of sABE was compared them with ABE8e in mammalian cells. Our findings indicate that sABE has high fidelity but reduced activity compared with 8e-SaCas9-KKH. But SpCas9-based sABE also holds great potential for further optimization, as SpCas9 has recently developed a near-PAMless variant, SpRY [29]. Overall, our study provides a comprehensive analysis of sABE and characterizes it as a versatile and robust genome-editing tool for in vivo applications.

To overcome the size limitation of AAV vectors, one approach is to split SpCas9 into two parts and package them into separate AAVs. Previous studies have demonstrated that SpCas9 and SaCas9 can be split into two fragments and still enable in vivo base editing [30–32]. Half of each base editor is fused to half of a fast-splicing split intein, following a co-infection by AAV particles expressing each base editor-split-intein half, protein splicing in trans reconstitutes full-length base editor. However, dual vector system is more expensive to produce and more sophisticated to titrate stoichiometrically compared to a single vector system. Another strategy is to search for smaller CRISPR-Cas effectors. Small base editor variants that fit within a single AAV have also been developed and used for in vivo applications [33, 34]. However, small Cas9 orthologs usually have restrictive PAM. Removing domains from gene editors that retain function both in vivo and in vitro is also considered to be a feasible strategy [13, 14, 35]. The size of the sABE system has been remarkably reduced after domain deletion, but its activity is severely reduced, yet it still works effectively together with ABE8e. We observed that sABE system has moderate editing in vivo after packaging it into one AAV vector. This can be attributed to the large genome size (4.83 kb), which exceeds the packaging limit of 4.7 kb for AAVs. Furthermore, previous study showed that CjCas9 expression was barely detectable in cells transfected with AAV-miniCMV-CjCas9 plasmid by western blot [36]. Thus, we speculate that the miniCMV promoter may also negatively impact on sABE expression. Nonetheless, it holds promise for future optimization, such as introducing additional mutations to restore enzyme function, placing the U6-sgRNA component in the antisense direction, or adding additional regulatory elements for enhanced protein expression [14, 30].

Surprisingly, CBE deletion variants displayed lower efficiency in editing endogenous sites compared to exogenous sites. We propose that the reduction was caused by the lack of target strand-nicking after domain deletions that initiates long-patch BER. The deaminated strand is preferentially used as a template for repair, and CBE relies more on it [2]. To increase the efficiency, we speculated that exchanging APOBEC1 with a more active deaminase would be beneficial. The PE system requires the strand-nicking activity to cleave the non-target strand, allowing the 3′ DNA end to hybridize and reverse transcribe the template on the pegRNA [4]. However, further studies are needed to determine whether domain deletions would affect the nicking activity of PE.

Previous studies have demonstrated that the removal of conserved SaCas9 REC and HNH functional domains still maintains DNA binding activity. Furthermore, the tripartite VPR (VP64-p65-Rta) activation domain fused with miniSaCas9 has shown efficient transactivation activity [37]. However, 8e-SaCas9-KKH has not been able to adapt to the deletion strategy, possibly due to differences in protein structures between SaCas9 and SpCas9. This indicates that deletion strategy may not be applicable to all Cas effectors.

The removal of HNH and REC2 domains in sABE leads to broader editing window (A3-A15). However, this wider range of editing may also lead to additional bystander mutations. Our previous research demonstrated that eliminating the linker in ABE8.17 result in efficient base editing within a narrowed window (2–4 nts) in human HEK293FT cells [38]. Similarly, in the present study, sABE was observed to have a narrow editing window when the same linker was removed (Additional file 1: Fig. S11). However, the sABE without such linker displayed lower editing efficiency, indicating that additional mutations might need to be introduced for further optimization between editing efficiency and window.

## Conclusions

In summary, we have developed an sABE system that enables efficient in vivo genome editing with a broadened editing window and high fidelity. Future efforts may focus on refining the sABE system through directed protein evolution or rational protein engineering strategies to improve editing specificity and efficiency. Overall, the sABE system represents a promising tool for both basic research and clinical therapeutics.

## Methods

### Plasmid construction

The ABE8e, ABE8.17, AncBE4max, and PE plasmids were obtained from Addgene (#138,489, #136,298, #138,270 and #136,463). mCherry-T2A-GFP was described in detail in our previously published study [39]. The DNA fragments of deletion variants from ABE, CBE and PE were used for in-fusion cloning by ClonExpress Ultra One Step Cloning Kit (Vazyme, Nanjing, China). All PCR primers are listed in Additional file 1: Table S2 and were synthesized by Sangon Biotech. The pegRNA and sgRNA were synthesized by Genscript Biotech (Nanjing).

### Cell culture and transfection

HEK293T and N2a cell lines were cultured in Dulbecco’s modified Eagle’s medium (DMEM) (Meilun Biotechnology Co., Ltd) supplemented with 10% fetal bovine serum (HyClone, China) and incubated at 37 °C in an atmosphere of 5% CO_2_. The cells were seeded into six-well plates and transfected using Hieff TransTM Liposomal Transfection Reagent (Yeasen, Shanghai, China) following the manufacturer’s instructions. After 72 h, the cells were collected and used for genotyping by EditR [40]. All the primers used for genotyping are listed in Additional file 1: Table S4-S5.

### mCherry/EGFP reporter assay

HEK293T cells in a six-well plate were transfected with 750 ng gRNA, 750 ng mCherry/EGFP reporter, and 1500 ng of each variant. The fluorescent images were imaged with a microscope (*Olympus* DP74). Cells were harvested following 72 h of incubation for editing quantification by flow cytometry.

### mRNA and gRNA preparation

The sABE plasmids were linearized with NotI and transcribed in vitro using the HiScribe T7 ARCA (Anti-Reverse Cap Analog) mRNA kit (NEB). mRNA was purified using the RNeasy Mini Kit (QIAGEN) according to the manufacturer’s protocol. The sgRNA was synthesized by Genscript Biotech (Nanjing).

### AAV8 production and injection

AAV8 containing the sABE elements targeting *Pcsk9* Exon6 SA was obtained from Genomeditech (Shanghai, China). For AAV vector injections, 8-week-old female ICR mice were injected with 4 × 10^12^ GCs per mouse via the tail vein. Mice were euthanized 30 days after vector administration, and liver tissues were collected for analysis.

### Microinjection of mouse zygotes and genotyping

Briefly, a mixture of mRNA (50 ng/μl) and sgRNA (30 ng/μl) was co-injected into the cytoplasm of pronuclear-stage zygotes. Each group was injected with an average of approximately 20 zygotes to test the base editing efficiency. The injected zygotes were transferred to potassium simplex optimized medium (KSOM) for culture at 37 °C, 5% CO_2_, and 100% humidity. Then, the injected single zygote was collected at the blastocyst stage. Genomic DNA was extracted in embryo lysis buffer 1% NP40 (Meilun Biotechnology Co., Ltd) at 56 °C for 60 min and then at 95 °C for 10 min in a Bio-Rad PCR Amplifier. Then, the extracted products were amplified by PCR (95 °C, 5 min for pre-degeneration, 42 cycles of [95 °C, 30 s; 58 °C, 30 s; 72 °C, 30 s] 72 °C, 5 min for extension) and determined by Sanger sequencing. All primers used for genotyping are listed in Additional file 1: Table S5.

### Statistical analysis

All data are expressed as means ± S.E.M. of at least three individual determinations for all experiments. Data were analyzed by Student’s *t*-test via the GraphPad prism software 8.0.1. The probability value smaller than 0.05 (*P* < 0.05) was statistically significant. *P < 0.05; **P < 0.01; ***P < 0.001; ****P < 0.0001.

## Supplementary Information

**Additional file 1: Fig. S1.** Screening single deletion for CBE. **Fig. S2.** Screening single deletion for PE. **Fig. S3.** Cas9 deletion variants cleavage activity. **Fig. S4.** Representative base editing pattern analyzed by EditR. **Fig. S5.** Comparison of editing efficiency in seven target sites using sABE and 8e-SaCas9-KKH. **Fig. S6.** Screening single deletion landscape for 8e-SaCas9-KKH. **Fig. S7.** sABE is inhibited by anti-CRISPR (Acr) proteins. **Fig. S8.** Comparison of editing efficiencies of 8e and sABE in HEK293T cell line. **Fig. S9.** In vivo genome editing of sABE via all-in-one AAV delivery. **Fig. S10.** Sanger sequencing chromatograms of sABE mediated base editing in mouse embryos. **Fig. S11.** Comparison of editing efficiencies of sABE and sABE-NL. **Table. S1.** Generation of targeted editing in mice embryos. **Table. S2.** Primers used for domain deletion variants construction. **Table. S3.** PegRNA sequence. **Table. S4.** Target sites used in HEK293T cells. **Table. S5.** Target sites used in N2a cells and mouse embryos.

**Additional file 2.** All data generated or analyzed during this study are included in this published article and its supplementary information file 2.

## Data Availability

The authors state that all data necessary for confirming the conclusions presented in this article are represented fully within the article or can be provided by the authors upon request. All data generated or analyzed during this study are included in this published article and its supplementary information files (Additional File 2 Source Data).

## Abbreviations

CBEs: Cytidine base editors
ABEs: Adenine base editors
PE: Prime editor
CGBEs: C-to-G base editors
AYBE: Adenine transversion base editor
CRISPR: Clustered regularly interspaced short palindromic repeat
SpCas9: *Streptococcus pyogenes* Cas9
SaCas9: *Staphylococcus aureus* Cas9
PAM: Protospacer adjacent motif
sgRNAs: Single guide RNAs
pegRNA: Prime editing guide RNA
sABE: ABE8e without REC2 and HNH domains
Acr: Anti-CRISPR
AAV: A single adeno-associated virus
*Pcsk9*: Proprotein convertase subtilisin/kexin type 9
*GAA*: Alpha glucosidase
MFN2: Mitofusin 2
*Ar*: Androgen receptor
*Hoxd13*: Homeobox D13
*TYR*: Tyrosinase
