# Supplementary material for: A new compact adenine base editor generated through deletion of HNH and REC2 domain of SpCas9
Source: BMC Biol. 2023 Jul 11;21:155. doi: 10.1186/s12915-023-01644-9 (PMC10337206; doi:10.1186/s12915-023-01644-9)
Supplement: Supplementary file 1 — Additional file 1: Fig. S1. Screening single deletion for CBE. Fig. S2. Screening single deletion for PE. Fig. S3. Cas9 deletion variants cleavage activity. Fig. S4. Representative base editing pattern analyzed by EditR. Fig. S5. Comparison of editing efficiency in seven target sites using sABE and 8e-SaCas9-KKH. Fig. S6. Screening single deletion landscape for 8e-SaCas9-KKH. Fig. S7. sABE is inhibited by anti-CRISPR (Acr) proteins. Fig. S8. Comparison of editing efficiencies of 8e and sABE in HEK293T cell line. Fig. S9. In vivo genome editing of sABE via all-in-one AAV delivery. Fig. S10. Sanger sequencing chromatograms of sABE mediated base editing in mouse embryos. Fig. S11. Comparison of editing efficiencies of sABE and sABE-NL. Table. S1. Generation of targeted editing in mice embryos. Table. S2. Primers used for domain deletion variants construction. Table. S3. PegRNA sequence. Table. S4. Target sites used in HEK293T cells. Table. S5. Target sites used in N2a cells and mouse embryos. [file 12915_2023_1644_MOESM1_ESM.docx]

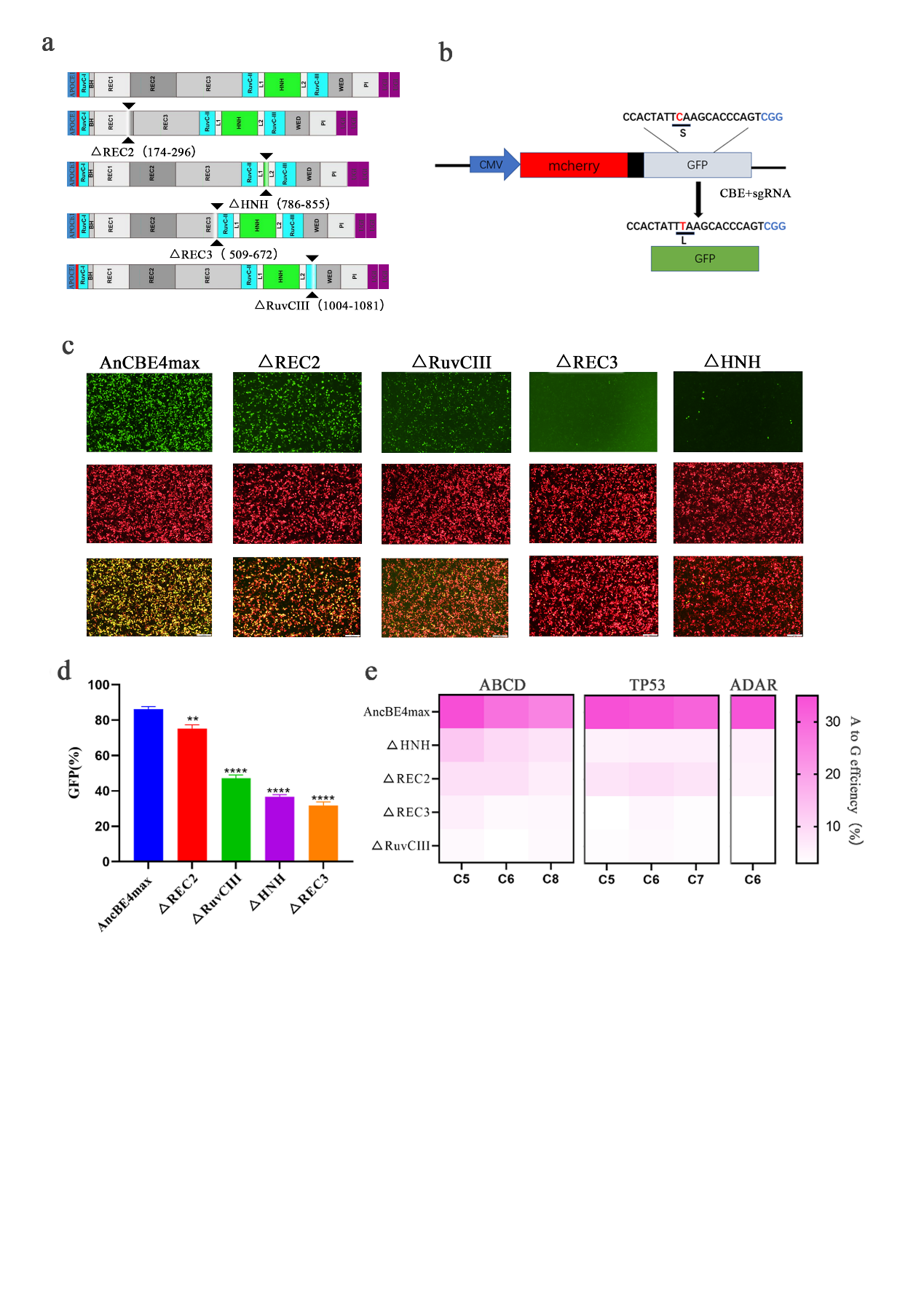


**Fig. S1 Screening single deletion for CBE. a** Schematic of domain deletion of AncBE4max. **b** Schematic of mCherry/EGFP reporter. **c** Representative fluorescence microscopy images of deletion variants analysis. **d** Comparison of editing efficiency of AncBE4max deletion variants by flow cytometry analysis (FCA). **e** Comparison of editing efficiency of AncBE4max deletion variants in HEK293T cell line by plasmid transient transfection (n = 3).


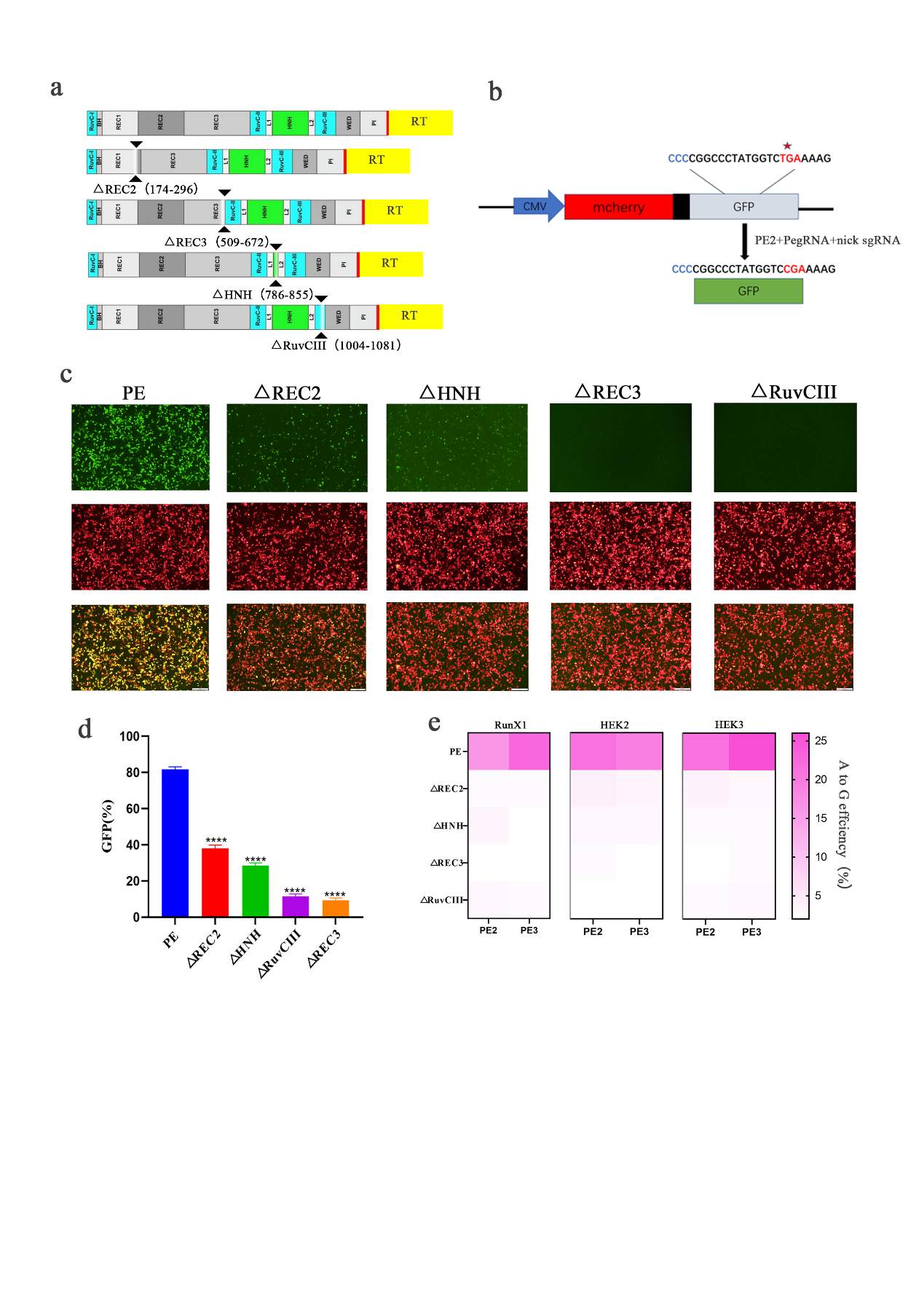


**Fig. S2 Screening single deletion for PE. a** Schematic of domain deletion of PE. **b** Schematic showing the mCherry/EGFP reporter. **c** Representative fluorescence microscopy images of deletion variants analysis. **d** Comparison of editing efficiency of PE deletion variants by flow cytometry analysis (FCA). **e** Comparison of editing efficiency of PE deletion variants in HEK293T cell line by plasmid transient transfection (n = 3).


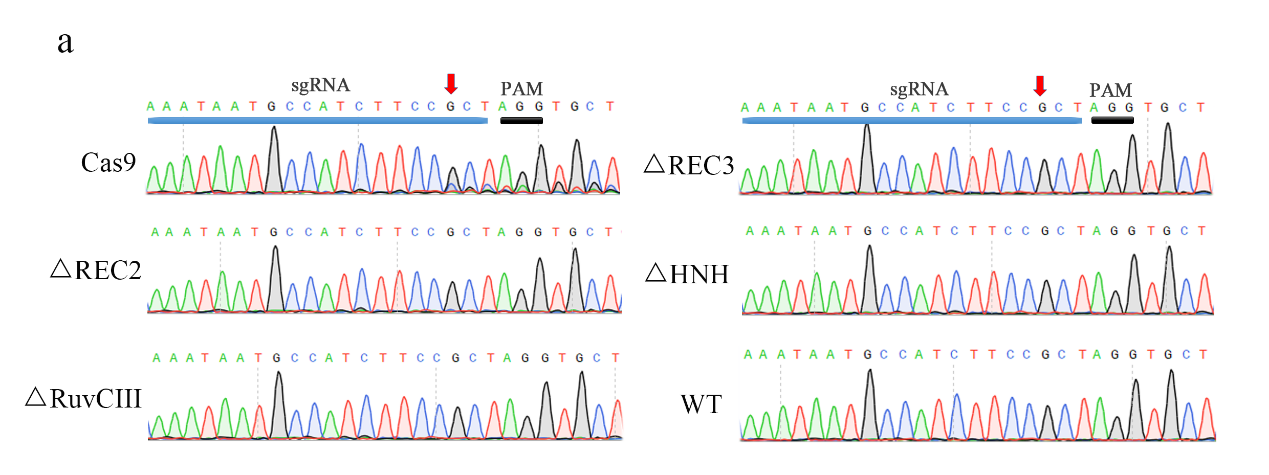


**Fig. S3** **Sanger sequencing chromatograms of Cas9 deletion variants cleavage activity.** The PAM sequence and spacer sequence of sgRNA are underlined in blue and black, respectively. Red arrows indicate the cleavage site.


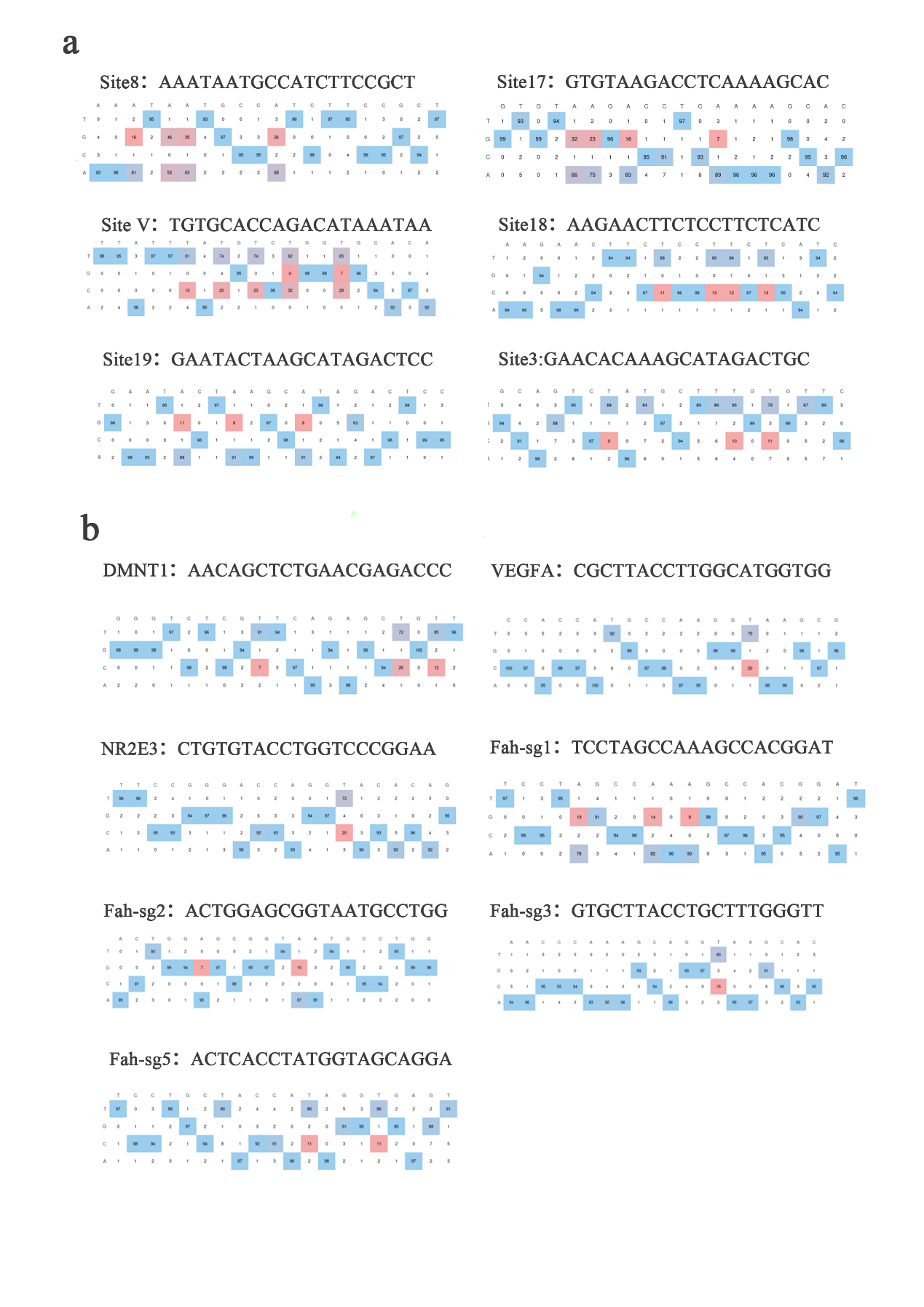

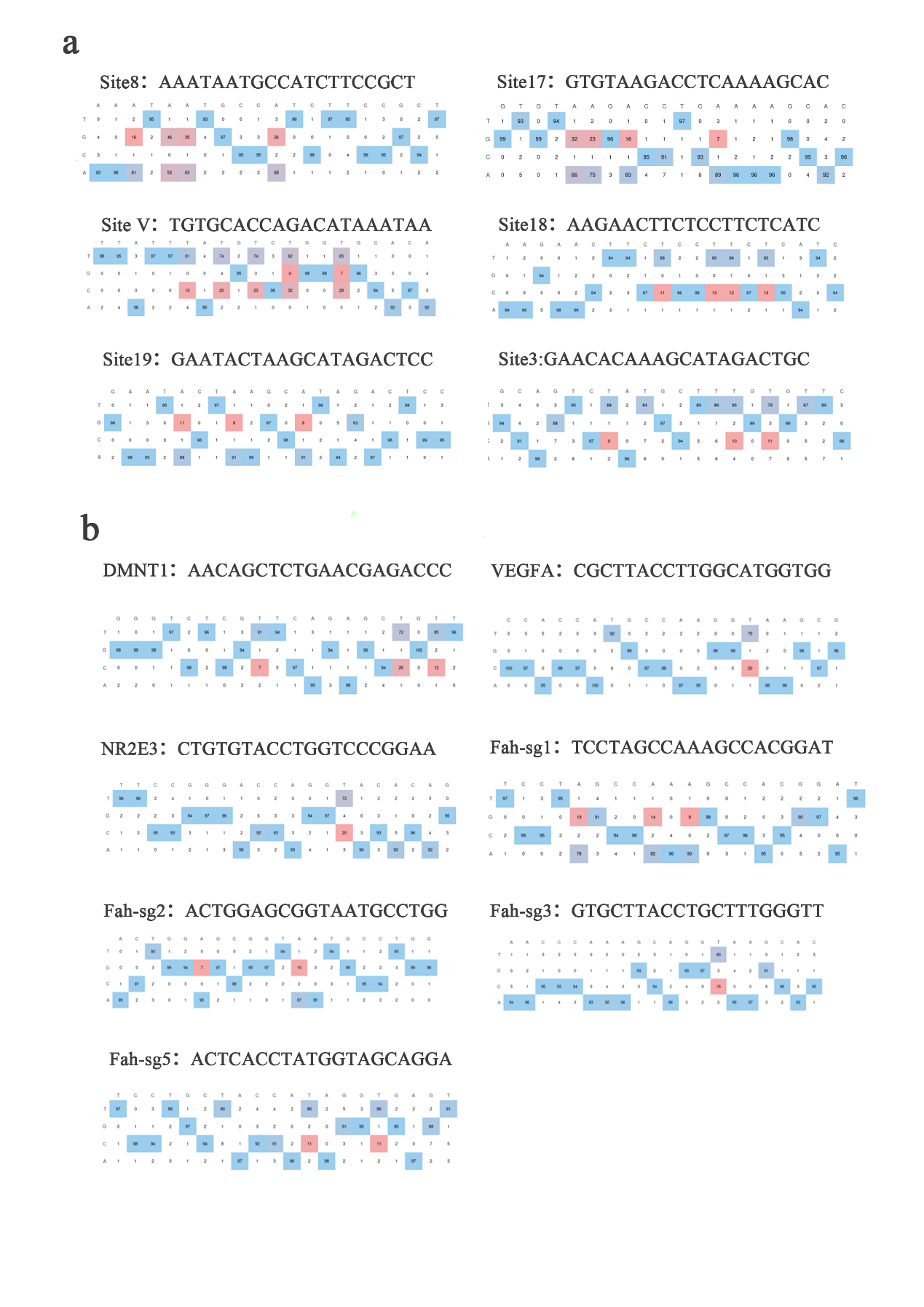


**Fig. S4** **Representative base editing pattern analyzed by EditR of 6 loci in HEK293T cells and 7 loci in N2a cells using sABE.**


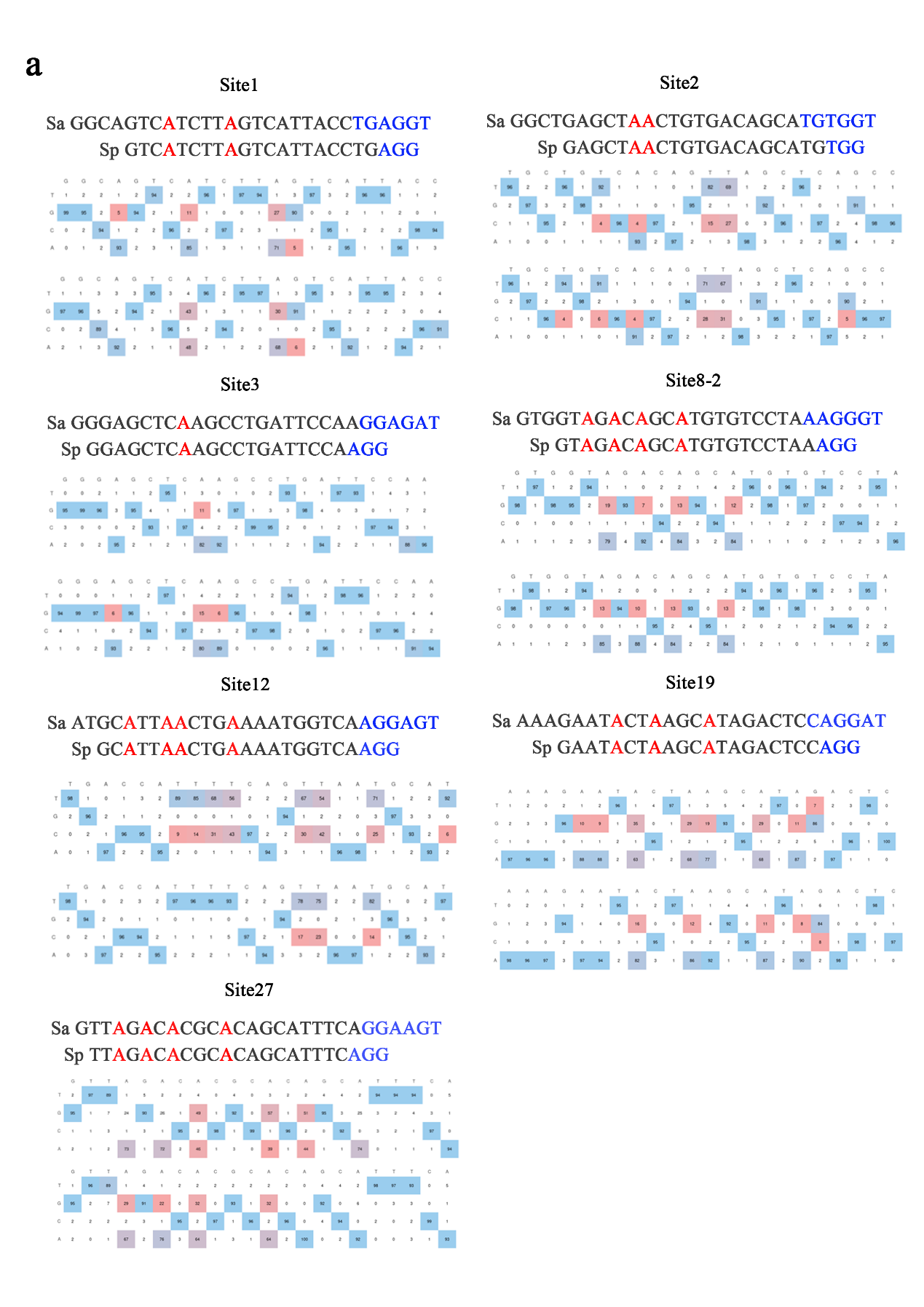


**Fig. S5** **Representative base editing pattern analyzed by EditR of 7 loci in HEK293T cells using 8e-SaCas9-KKH and sABE.** PAM region (blue) and target sites (red).


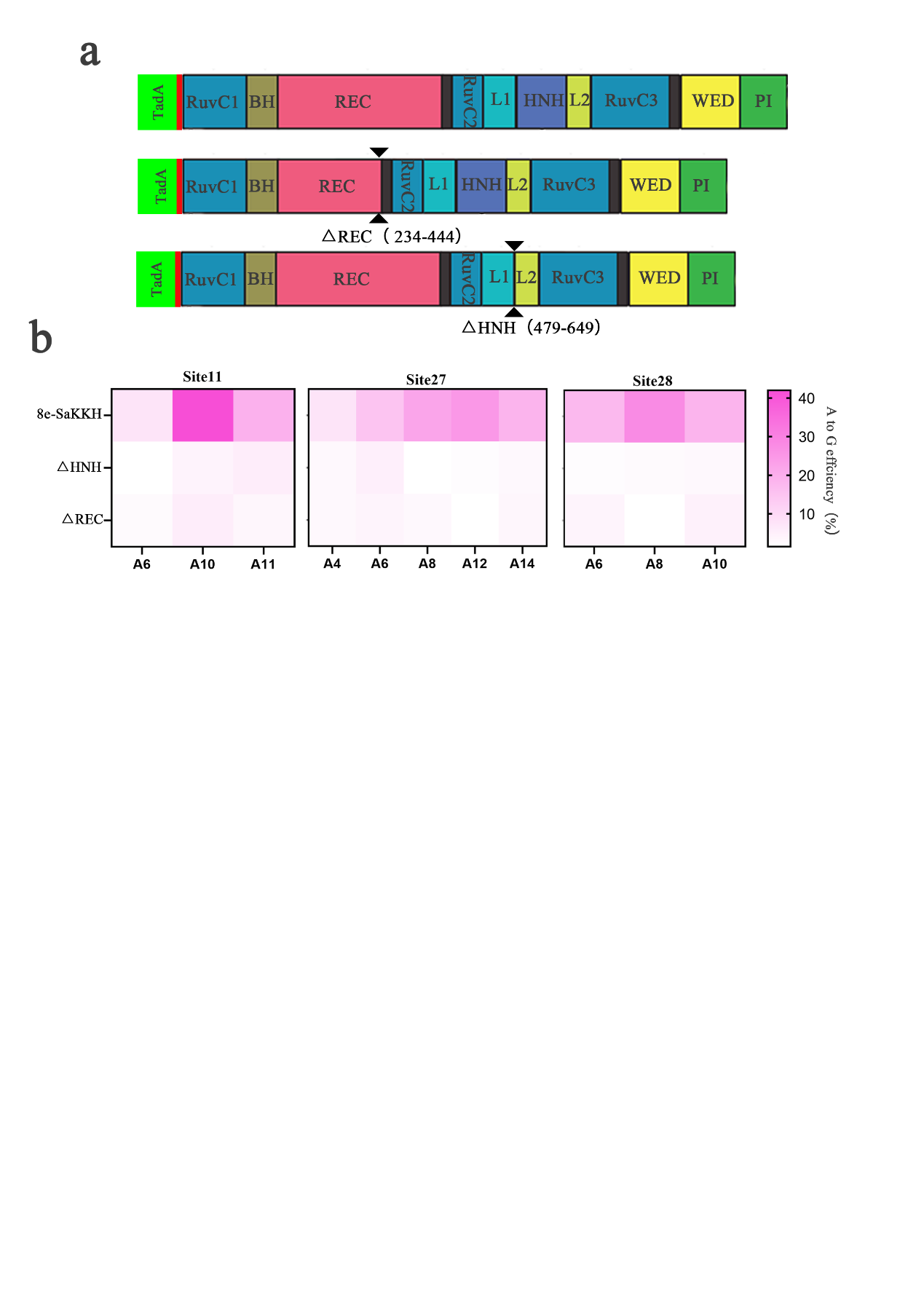


**Fig. S6 Screening single deletion landscape for 8e-SaCas9-KKH. a** Schematic of domain deletion of 8e-SaCas9-KKH. **b** Comparison of editing efficiencies of 8e-SaCas9-KKH deletion variants in HEK293T cell line by plasmid transient transfection (n = 3).


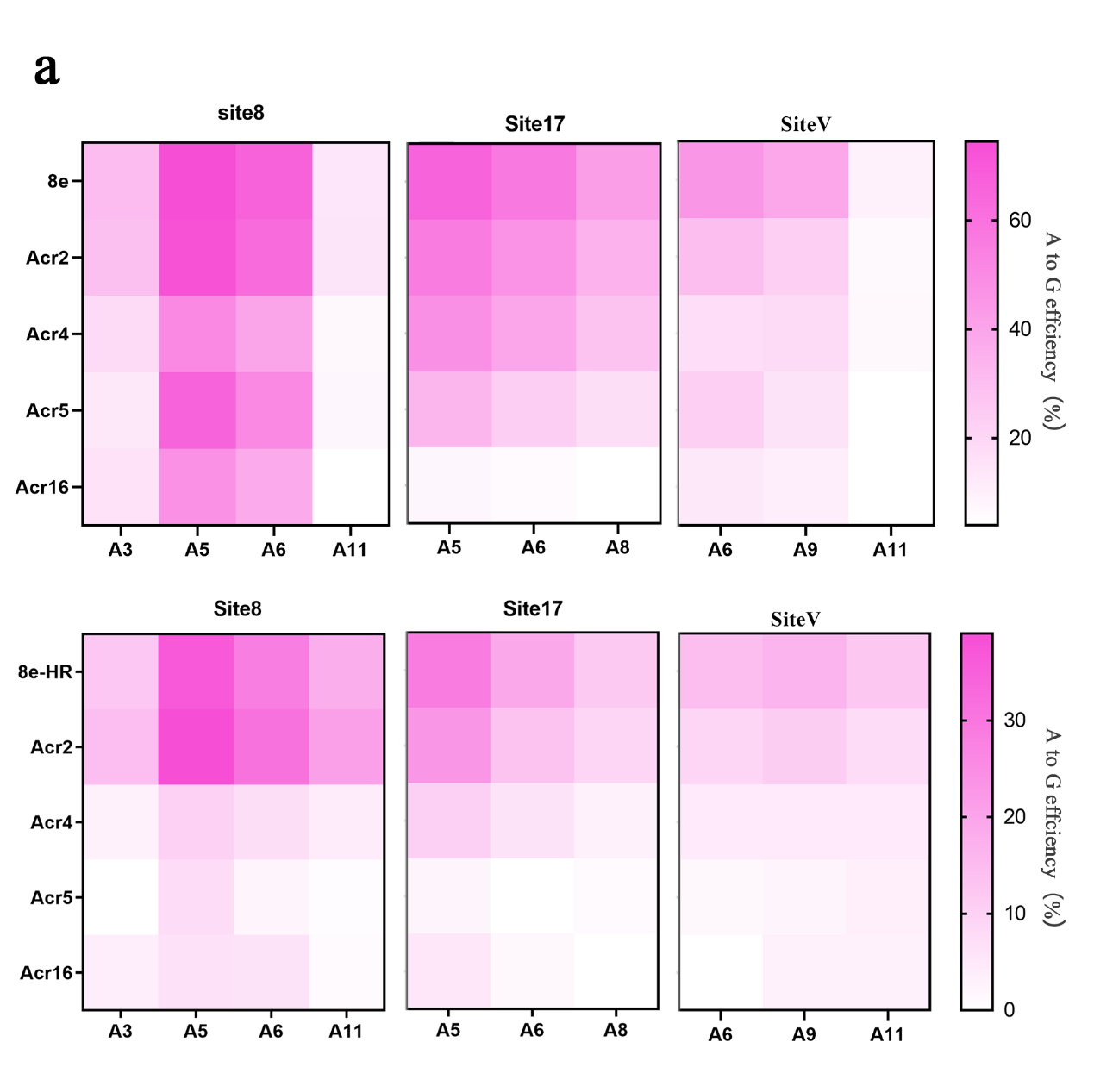


**Fig. S7** **sABE is inhibited by anti-CRISPR (Acr) proteins in human cells.** Genome editing of sABE in the presence of the 4 previously described Acrs. Plasmids expressing sABE, sgRNA, and each Acr (1:1:1) were co-transfected into HEK293T cells.


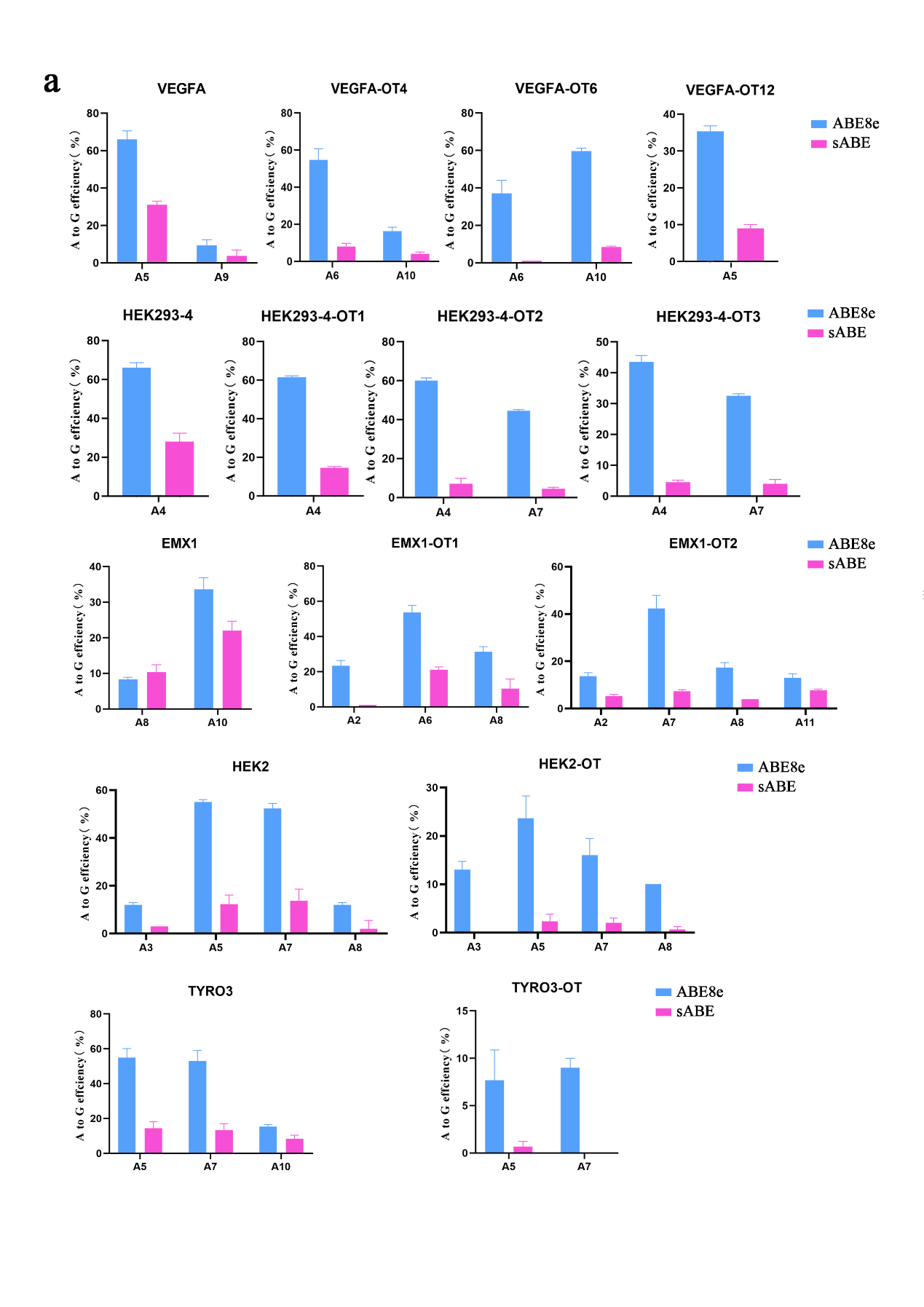


**Fig. S8 Comparison of editing efficiencies of 8e and sABE in HEK293T cell line by plasmid transient transfection (n = 3).**


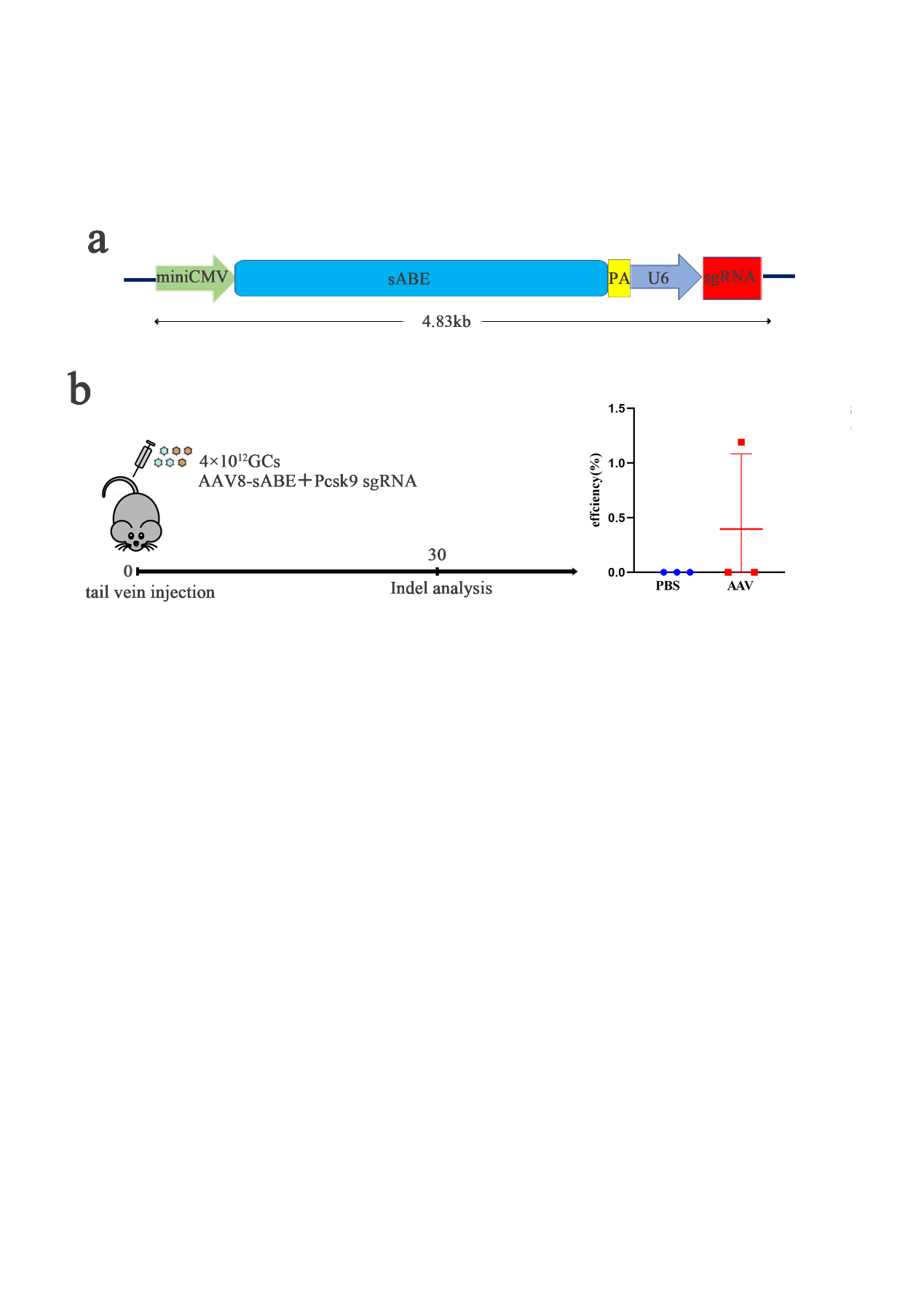


**Fig. S9** **In vivo genome editing of sABE via all-in-one AAV delivery. a** Schematic of the AAV vectors. sABE and the sgRNA were packaged into AAV8 particles. The miniCMV promoter is highlighted in cyan, sABE in blue, short translation terminator in yellow, U6 promoter in grey and sgRNA in red, respectively. **b** Experimental timeline of AAV-sABE tail-vein injections, and editing efficiency analyses.


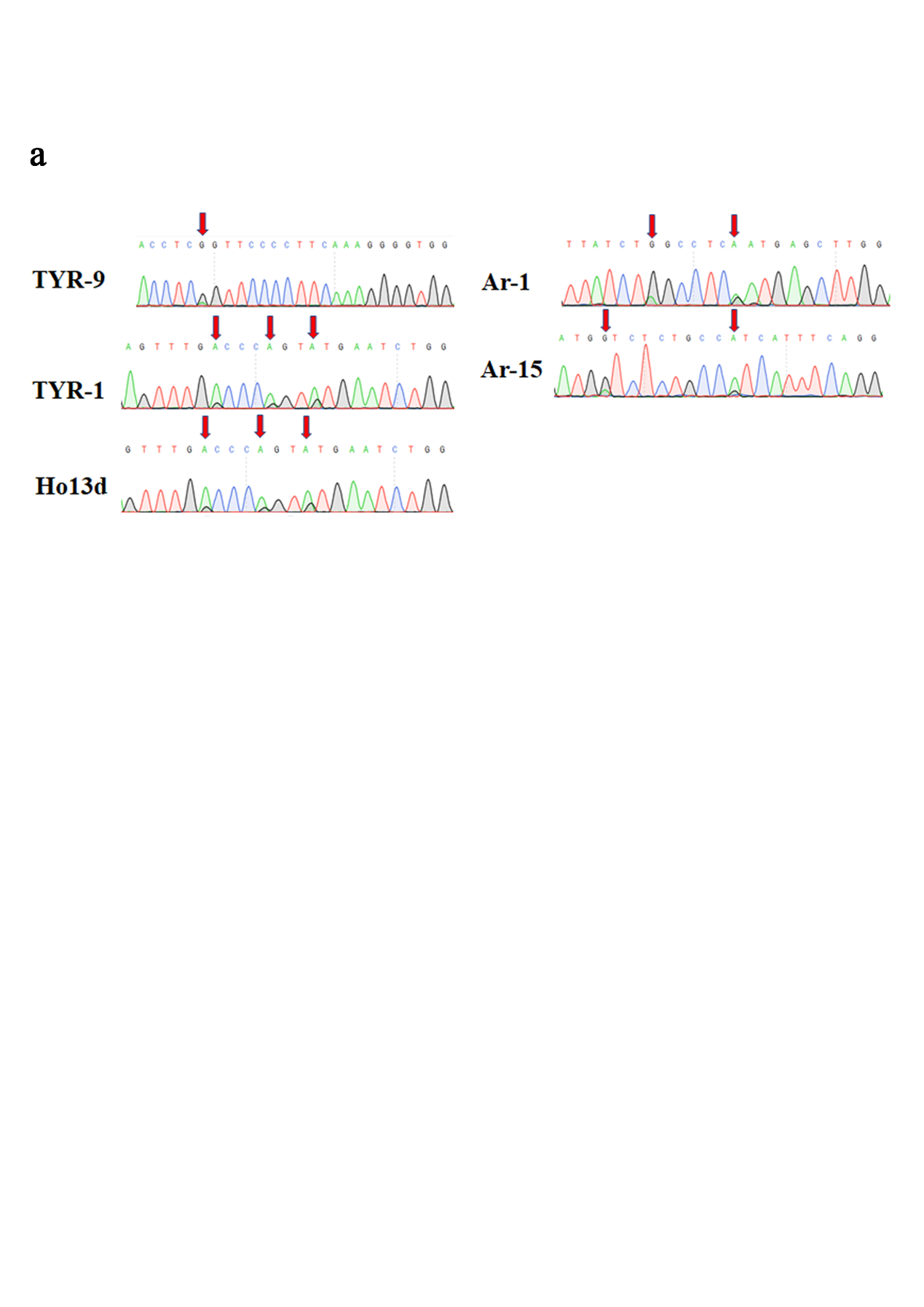


**Fig. S10** **Sanger sequencing chromatograms of sABE mediated base editing in mouse embryos.** Red arrows indicate the targeted bases.


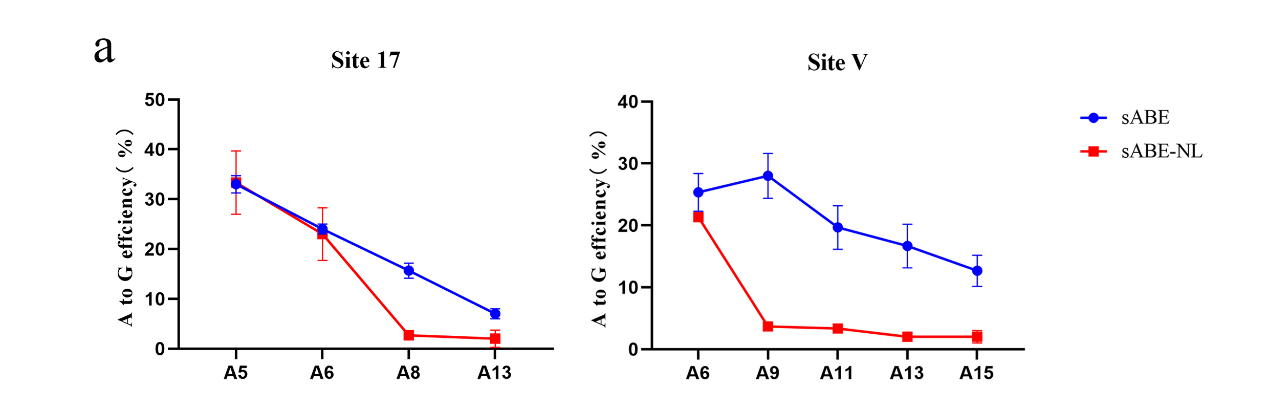


**Fig. S11** **Comparison of editing efficiencies of sABE and sABE-NL in HEK293T cell line by plasmid transient transfection (n = 3).**

**Table. S1** **Generation of targeted editing in mice embryos in this study.**

| **Target site** | **No. of blastocysts** | **No. of mutants (%)** | **Average frequencies of editing (%)** |
| --- | --- | --- | --- |
| ***Tyr*-9** | 13 | 10 (76.9%) | 65.2±13.14% |
| ***Tyr*-1** | 11 | 6 (55%) | 17±12.6% |
| ***Ar*-15** | 16 | 14 (87.5%) | 46.9±12.8% |
| ***Ar*-1** | 20 | 7 (35%) | 40.4±19.4% |
| ***Ho13d*** | 19 | 4 (21%) | 16.3±3.1% |

**Table. S2** **Primers used for one step coining domain deletion variants construction in this study.**

| **Deletion domain** | **Fragment** | **Vector** |
| --- | --- | --- |
| Sp-del HNH | F:atgaagcggatcgaactgctgaacgccaagct  R:tattgctgataaatctggagccggtga | F:gatttatcagcaataaaccagccagcc  R:ttcgatccgcttcattctctcgc |
| Sp-del REC2 | F:ctgatcgagggcgactccgacgccatcctgctga  R:ggtgatgatgaccggttagactttcct | F:ccggtcatcatcaccatcacca  R:gtcgccctcgatcaggaagtg |
| Sp-del REC3 | F:atcgtgctgaccctgaagctgatcaacggcatcc  R:ataccaaacgacgagcgtgacaccacg | F:ctcgtcgtttggtatggcttcattca  R:cagggtcagcacgatatct |
| Sp-del RuvCI | F:agcatcggcctggccgccgaggccacccggc  R:ggtgatgatgaccggttagactttcct | F:ccggtcatcatcaccatcacca  R:ggccaggccgatgctgtacttcttgtc |
| Sp-del RuvCII | F:gatagcctgcacgagggacagaagaacagcc  R:tattgctgataaatctggagccggtga | F:gatttatcagcaataaaccagccagcc  R:ctcgtgcaggctatcgccctggccgg |
| Sp-del RuvCIII | F: aaaagtaccctaagaccgtgcggaaagtgc  R:tattgctgataaatctggagccggtga | F:gatttatcagcaataaaccagccagcc  R:cttagggtactttttgatcagggcgg |
| Sa-del HNH | F:ataattatcgagcttcggtacgccacgagaggc  R:catggtaatagcgatgactaatacgtagatgtact | F:atcgctattaccatggtgatgcggttt  R:aagctcgataattatat |
| Sa-del REC2 | F:atgggacattgtacaaaaaaagtggatctgtca  R:ggccgccggccttttaccttttttaatgatttgtgggtgcttct | F:aaaaggccggcggccacgaaaaagg  R:tgtacaatgtcccataagcatctcg |

**Table. S3** **PegRNA used in this study.**

| HEK2 pegRNA | gggcagacgtcctgccactggttttagagctagaaatagcaagttaaaataaggctagtccgttatcaacttgaaaaagtggcaccgagtcggtgcagcccaatcctcagtggcaggacgtct |
| --- | --- |
| HEK3 pegRNA | ggcccagactgagcacgtgagttttagagctagaaatagcaagttaaaataaggctagtccgttatcaacttgaaaaagtggcaccgagtcggtgctggaggaaccagggcttcctttcctctgccatcacgtgctcagtctg |
| Runx1 pegRNA | gcattttcaggaggaagcgagttttagagctagaaatagcaagttaaaataaggctagtccgttatcaacttgaaaaagtggcaccgagtcggtgctgtctgaagcaatcgcttcctcctgaaaat |

**Table. S4** **Target sites used in HEK293T cells.**

| **Target Site** | **Target site sequence (5’-3’)** | **Primers** |
| --- | --- | --- |
| ADAR | caaatctgtcacattgggtaagg | F:agaaggcacaatccctgtgg  R:cctgtgtttgcaccaaatgct |
| ABCD1 | ggagccacaggagccgctgcagg | F:ggagcccacaaagtctaccc  R:ctgggagaagtagaggcggt |
| TP53 | ccttcccagaaaacctaccaggg | F:ctgtccccggacgatattga  R:tgatgggatggataaaagccca |
| HEK2-nick | tgccattctaccaacaatagagg | F:cacaggctaccccctaagtc  R:ccctcagcattcagccactaa |
| HEK3-nick | gcacatactagcccctgtctagg | F:gggaaacgcccatgcaatta  R:ggtgccctgagatcttttcct |
| Runx1-nick | atgaagcactgtgggtacgaagg | F:agatgtagggctagaggggtg  R:tcacaaacaagacagggaactg |
| Site8 | aaataatgccatcttccgctagg | F:gcgagggttacagttctgtttag  R:gcctcctgctgtcattgatatt |
| Site17 | gtgtaagacctcaaaagcacagg | F:ggagaaccataggcagaagaat  R:ctactgagttgcttgagctctta |
| Site19 | gaatactaagcatagactccagg | F:agaaggtaagtgcatggtaagg  R:ccagatttctcagcctctttct |
| Site3 | gaacacaaagcatagactgcggg | F:actgccattctaccaacaataga  R:aagcaggtgattacaggattga |
| Site18 | gatgagaaggagaagttcttagg | F:caccccttcagtccatgctt  R:tctgatggggaggaacgagt |
| SiteV | tgtgcaccagacataaataatgg | F:gctttctgccataatgaagtctg  R:aaagagggctgaacacaatcta |
| ATM | gtacctgaatgattcctgcctgg | F:Tttgtatggctgtggtggag  R:tttcctgtgtctccctgaattt |
| LIPA | cttcctgcaacatggcttgctgg | F:Acaacttcagagttaccacctatc  R:taccttgccagtgctgttt |
| MTMI | gttattctccaatggtgattggg | F:ggattattgatttggcacccttatt  R:tctccagtctggtatggtactt |
| SCNN1G | gtgctgtacctgccaaggtgggg | F:cctcactgtggctggtttat  R:gggagaatctaggctgctttc |
| SERPINA1 | ggccattgccggtggtcagctgg | F:agctccttgaccaaatccac  R:ccagtccaacagcaccaata |
| TH | cgcctcacccttgggccccgcgg | F:cttgtccagctctgacactt  R:tctccttctctctctcttcctg |
| Sp-site1 | gtcatcttagtcattacctgagg | F:tgttcacccagtacctacca  R:ccatagcacttcccttccaaata |
| Sa-site1 | ggcagtcatcttagtcattacctgaggt |  |
| Sp-site2 | gagctaactgtgacagcatgtgg | F:actgccattctaccaacaataga  R:cctcagcattcagccactaata |
| Sa-site2 | ggctgagctaactgtgacagcatgtggt |  |
| Sp-site3 | ggagctcaagcctgattccaagg | F:ggctgaccaagagtgaagaa  R:ccctcctctgcgtgaattt |
| Sa-site3 | gggagctcaagcctgattccaaggagat |  |
| Sp-site8-2 | gtagacagcatgtgtcctaaagg | F:agtctcctgcttctctgtgata  R:ataggaggcagaggtgtgaa |
| Sa-site8-2 | gtggtagacagcatgtgtcctaaagggt |  |
| Sp-site12 | gcattaactgaaaatggtcaagg | F:tgtgggcaataatgctctgata  R:agtcccagtcctagttcctc |
| Sa-site12 | atgcattaactgaaaatggtcaaggagt |  |
| Sa-Site19 | aaagaatactaagcatagactccaggat |  |
| Sp-site27 | gttagacacgcacagcatttcaggaagt | F:gagactgattgcgtggagtt  R:gaggtcagaagtttgagaccag |
| Sa-Site27 | ttagacacgcacagcatttcagg |  |
| Sp-site8-2M1 | ggataatgccatcttccgctagg |  |
| Sp-site8-2M2 | aagcaatgccatcttccgctagg |  |
| Sp-site8-2M3 | aaatagcgccatcttccgctagg |  |
| Sp-site8-2M4 | aaataatatcatcttccgctagg |  |
| Sp-site8-2M5 | aaataatgctgtcttccgctagg |  |
| Sp-site8-2M6 | aaataatgccactttccgctagg |  |
| Sp-site8-2M7 | aaataatgccatcccccgctagg |  |
| Sp-site8-2M8 | aaataatgccatcttttgctagg |  |
| Sp-site8-2M9 | aaataatgccatcttccattagg |  |
| VEGFA | ggtgagtgagtgtgtgcgtgtgg | F:gtgcagacggcagtcactagg  R:tattggaatcctggagtgaccc |
| VEGFA-OT4 | gagtgagtgagtgtgtgtgtggg | F:gagggacaagaactacaagtctc  R:tcatcgttatcgctcatttcct |
| VEGFA-OT6 | tgtgagtaagtgtgtgtgtgtgg | F:agatgtttggtgggactgatg  R:ctgtgtcttccttctgctgtt |
| VEGFA-OT12 | ggtgagtgtgtgtgtgcatgtgg | F:tcagcaatgcttatattactggc  R:gcgctttccctttgctagaatc |
| HEK293-4 | ggcactgcggctggaggtggggg | F:aacccaggtagccagaga  R:gaacggagacacacacaca |
| HEK293-OT1 | tgcactgcggccggaggaggtgg | F:agccacaaggcagagaaag  R:ctcctcggagtcctcaagtat |
| HEK293-OT2 | ggcacgacggctggaggtggggg | F:gacacgggttgcctttct  R:gaagatggtgctttcccttcta |
| HEK293-OT3 | ggcatcacggctggaggtggagg | F:gggaggaaacagaactgagaag  R:gttgggcgcagagtagataag |
| EMX1 | gagtccgagcagaagaagaaggg | F:cagctcagcctgagtgttga  R:tcgtgggtttgtggttgc |
| EMX1-OT1 | gagttagagcagaagaagaaagg | F:gcacatgtatgtacaggagtca  R:gcttgtccatgtctaggaaaga |
| EMX1-OT2 | gagtctaagcagaagaagaagag | F:caatgtgcttcaacccatcacggc  R:catgaatttgtgatggatgcagtctg |
| HEK2 | gaacacaaagcatagactgcggg | F:ggacgtctgcccaatatgtaa  R:caagtgagaagccagtggaata |
| HEK2-OT | gaacacaatgcatagattgccgg | F:gccagagttaagaagttggagat  R:gcatgggcacatgtgtttg |
| TYRO3 | ggccacactagcgttgctgctgg | F:tgccctactggttgcttattt  R:cctgtgttcttcctgctaagg |
| TYRO3-OT | ggccacactagtgttgccgctgg | F:cggagcaggcaggtaaag  R:ctgctccctctccatttgtt |
| GAA | tgtagtccaggtcgttccactgg | F:caggtggtggagaacatgac  R:cgatatcacaaagaccctcagaa |
| MFN2 | gctggcccatgccctccaccagg | F:aagaagaaagggtagcagatgg  R:ttaagcagagcaaggatggg |

**Table. S5** **Target sites used in N2a cells and mouse embryos.**

| **Target Site** | **Target site sequence (5’-3’)** | **Primers** |
| --- | --- | --- |
| Dnmt1 | aacagctctgaacgagaccccgg | F:ccttcgggcatagcatggtc  R:tatatgcctcggcatcggtcc |
| NR2E3 | ctgtgtacctggtcccggaaagg | F:aactccaaacctcctcctct  R:acacatctttggctagcatctt |
| VEGFA | cgcttaccttggcatggtggagg | F:cctccgaaaccatgaactttct  R:gctcggcccagatcgta |
| Fah-1 | tcctagccaaagccacggattgg | F:cgcctagctgttctctctttat  R:tgatacaatgtcccacctcatc |
| Fah-2 | actggagcggtaatgcctggtgg | F:gcatcgggtcatctagattcttac  R:cagctccatccttccacttatg |
| Fah-3 | gtgcttacctgctttgggtttgg | F:agacctaagcttgctgtcttt  R:agcatcaccatgtctcagtatc |
| Fah-5 | actcacctatggtagcaggaagg | F:ccaacttcctccatctctacatc  R:gctggcagtcctagatttctt |
| 1-SD | cccataccttggagcaacggcgg | F:caggacgaggatggagattatg  R:ctcccgatgccactttactt |
| 4-SA | ggaagatggaagcagccaggtgg | F:acctcctggttctgtcatttac  R:agagtcacactcacctgtct |
| 6-SA | ttgcaggcctggagtttattcgg | F:aggagctggaggaggaatgtat  R:cgttgaggatgcggctatac |
| 7-SD | cctacctctggagcagaagctgg | F:gcctggagtttattcggaagag  R:ggacagaagagggactggataa |
| 8-SD | atgccaggtcatcacagtcgggg | F:tcacaggtgatgggatttcag  R:ctatggtggcaggatgatgg |
| 10-SD | gactcctacctgtgagaacatgg | F:gtgctctttctcactggttct  R:ccaccatcatcaccctcttt |
| 11-SA | ttctaggctgcagcttccattgg | F:tactcggagctgtctctcttt  R:gagatcccatgctccttgattt |
| TYR-9 | acctcagttccccttcaaagggg | F:agtctgtgacactcattaacctatt  R:ggtgttgacccattgttcattt |
| TYR-1 | agtttgacccagtatgaatctgg | F:tgccagaaagctgaatgatact  R:cttcgcttccctttcccttt |
| Ar-15 | atgatctctgccatcatttcagg | F:gagaattctaaggtgggagaagag  R:gcagagaagtagtgcagagttat |
| Ar-1 | ttatctagcctcaatgagcttgg | F:catcccagaagatgactgtatca  R:ggcccaaagaaccattcaataa |
| Hoxd13 | gtttcagaatcgaagggtgaagg | F:caccaaactgcagctcaaag  R:agaaccagataaaggtcaagagag |
